# The Role of Worldview in Moral Case Deliberation: Visions and Experiences of Group Facilitators

**DOI:** 10.1007/s10943-021-01246-1

**Published:** 2021-04-08

**Authors:** Benita Spronk, Guy Widdershoven, Hans Alma

**Affiliations:** 1 Amsterdam UMC, Vrije Universiteit Amsterdam, De Boelelaan 1117, Postbus 7057, 1007 MB Amsterdam, The Netherlands; 2 Department of Ethics, Law and Humanities, Amsterdam UMC, Vrije Universiteit Amsterdam, De Boelelaan 1089 a, 1081 HV Amsterdam, The Netherlands; 3 Department of Religion and Theology, Vrije Universiteit Amsterdam, De Boelelaan 1105, 1081 HV Amsterdam, The Netherlands

**Keywords:** Moral case deliberation, Clinical ethics support, Worldview, Religion, Spirituality, Values

## Abstract

This study investigates the role of worldview in moral case deliberation (MCD). MCD is a form of clinical ethics support which aims to assist caregivers in reflection on moral dilemmas, experienced in daily practice. Bioethicists acknowledge that existential and religious aspects must be taken into account in the analysis of ethical questions, but it remains unclear how these elements are addressed in clinical ethics support. We investigated how facilitators of MCD address worldview in MCD. MCD facilitation is often done by spiritual caregivers, but not in their role as spiritual caregiver. Discussing worldview is no standard part of the procedure in MCD. This study was qualitative, focusing on the views and experiences of the facilitators of MCD. Semi-structured interviews (*N* = 12) were conducted with facilitators of MCD. Grounded theory was used for analysis. The results show that worldview plays both an explicit and an implicit role in the MCD process. The explicit role concerns the religious beliefs of patients and professionals. This calls for avoiding stereotyping and devoting attention to different visions. The implicit role comes to the fore in addressing core values and spiritual fulfillment. In order to clarify the fundamental nature of values, more explicit attention for worldview might be useful during MCD. However, this should be done with caution as the term ‘worldview’ might be interpreted by participants in terms of religious and personal beliefs, rather than as an invitation to reflect on one’s view of the good life as a whole.

## Introduction

Care professionals frequently face difficult dilemmas. Do you agree to terminate a pregnancy at the parents’ request if the baby will be born with a disability? Should you tell a patient that his condition is terminal if the family has asked you not to? Dilemmas like these involve perceptions of the value of life and the individual’s worldview. “Worldview refers to fundamental beliefs about life, death and suffering that structure people’s ideas on how life events are related.” (Littooij et al., 2016a, p.7). “Worldview is part of ‘global meaning’, a basic set of beliefs and goals that guide the way in which people give meaning to their lives. (Littooij et al., 2016a, b; Park, 2013a, b, p. 358.).” It concerns questions which touch upon the fundamentals of our existence, defining who we are and where we seek to belong (Alma, 2018, p. 45; Aerts et al., 2007, p. 5; Taves et al., 2018; Plante & McCreadie, 2019, p. 321). Moral case deliberation is about reflecting on making professional choices and treatment decisions. Reflection on underlying values and norms is important in order to be able to make responsible choices. This reflection takes place in moral case deliberation. Values and norms can be formed by belief systems and are determined by the meanings people give and visions they have on life, death and suffering. That is the reason we opted to define worldview as part of global meaning. Bioethicists acknowledge the importance of worldview in clinical ethics support, both in a general sense (Kørup et al., 2018; Mustafa, 2014; Turner, 2003; White et al., 2018) and in specific areas (Bandini et al., 2017; Mathieu, 2016; Mohamed & Noor, 2014). They emphasize that existential and religious aspects must be taken into account in the analysis of ethical questions in clinical practice. The existing literature tends to focus on identifying and defining the various elements of worldview. It remains unclear how these elements are, or should be, addressed by those involved in clinical ethics support.

Moral case deliberation (MCD) has been developed as a component of clinical ethics to help care providers make morally conscionable choices. An MCD session explores an ethical issue described by one of the participants and drawn from his or her personal experience. The deliberation is structured by a specific method and is led by an experienced facilitator (Stolper et al., 2016). MCD facilitation is often done by spiritual caregivers, but not in their role as spiritual caregiver. Reflection on ethics can be part of spiritual care. Facilitating MCD, however, requires specific skills and knowledge of methodologies. Many spiritual caregivers are interested in MCD and are trained as facilitator. However, not every spiritual caregiver is a trained facilitator. In their role as facilitator, they are trained to address values, but not worldview, as this is no standard part of the procedure in MCD. The MCD session generally takes place within the clinical department concerned. It is attended by departmental staff and representatives of other disciplines involved in the case under discussion.

In this article, we examine how MCD facilitators approach worldview as a component of clinical ethics. Facilitators are a source of experience and knowledge, how worldview is addressed in MCD. Our choice was to rely on their experiences. In essence, MCD entails reflection on the right thing to do. It, therefore, considers the perspectives of all persons involved in the situation and explores their personal norms and values. The investigation of these values can be accompanied by a reflection on existential aspects. What importance do MCD facilitators attach to such existential aspects? How do they use them to enhance the deliberation?

Our first research question is therefore: What is the role of worldview in MCD? The second research question is: How do MCD facilitators act in response to worldviews?

We begin with a brief overview of MCD based on the literature, followed by a description of our research method and results. This is followed by a discussion of those results and the authors’ conclusions.

## Moral Case Deliberation

Certain issues in healthcare practice can be perceived as morally problematic by healthcare providers. This concerns situations in which uncertainty occurs regarding what is right to do. These issues are apt for moral case deliberation (MCD). MCD is a structured method for investigating these moral issues. An MCD focuses on a case presented by one of the participants. This case must involve a concrete, personal experience from the past or present, not a hypothetical situation (Stolper et al., 2016). Participants in MCD in healthcare are often healthcare professionals (doctors, nurses, paramedics), but might also include managers, family members or even patients themselves. Under the guidance of a trained facilitator, the group will investigate the case.

The main purpose of MCD is not to arrive at a solution, but to foster critical reflection on the ethical issue at hand. Underlying values associated with the issue at stake in the case are scrutinized (Stolper et al., 2016). During MCD, participants explore what is important to themselves and other participants. The facilitator guides them in sharing and exchanging their moral considerations with each other. In this way, the issue is jointly examined and perspectives on the case are broadened. MCD is not about proposing statements or convincing an opponent, but about creating space to think about the case together. By exchanging various perspectives, a ‘fusion of horizons’ (Gadamer, 1960) among the participants can be achieved. The underlying aim is to search for common ground between one’s own and others’ experiential horizons, in order to understand one another better and develop a richer, more complete understanding of the situation.

To structure the discussion, the facilitator uses a specific conversation method. Several such methods have been developed (Van Dartel & Molewijk, 2014). A familiar option is the ‘dilemma method’ (Molewijk & Ahlzen, 2011; Stolper et al., 2016) in which a key step is the analysis of the case in terms of perspectives, values and norms. It is customary to produce a chart or table listing the perspectives of all persons involved in the case, known as the ‘stakeholders’. Cultural and religious norms and values can be part of personal perspectives. Our research concerns the extent to which this is addressed by facilitators and whether or not this is questioned by facilitators. The participants in the MCD session seek to identify the values which underpin those perspectives, the norms which serve to concretize the values, and possible courses of action. The norms and values concerned are the personal visions of the stakeholders. Those of stakeholders who are not actually present during the discussion, such as the patient or his family, can also be explored by the group by means of accounts provided by those who know them well (Widdershoven et al., 2016, p.73). Other deliberation methods also explicitly address values.

## Research Methodology

### Data Collection

This study forms part of a larger research project examining the relationship between MCD and tragic situations. In healthcare practice, care providers can be confronted by a tragic situation in which they must make decisions which will have far-reaching consequences. To what extent can MCD help them make those decisions? We investigate the role of MCD in dealing with tragic situations by looking at harm, worldview and emotions. This article focuses on the aspect of worldview.

Semi-structured interviews were held with a number of MCD facilitators who were asked to give examples of MCD sessions they had conducted and to briefly explain the process and outcomes. Facilitators using the dilemma method were asked about the role of worldview. Respondents who do not use the dilemma method were asked whether the aspect of worldview is incorporated into their favored approach and, if so, how.

The following criteria were used to select respondents:

- A minimum of 1 year’s experience in MCD facilitation.
- Currently working in healthcare (hospital or psychiatric clinic).
- Representative distribution in terms of gender, age, professional background and field of operation.

Twelve facilitators were interviewed: six male and six female. They represent a wide range of disciplines and include three medical specialists, one nurse manager, one paramedic, two clinical ethicists, two healthcare managers and three spiritual counsellors. The respondents have acted as facilitators with various groups. Six work in hospitals, three in mental healthcare, and three in both. The facilitators use (or have used) a range of MCD methods: eight use the dilemma method alongside other methods, while four use only alternative methods. A summary of characteristics of respondents is given in Table 1.

**Table 1:** Baseline characteristics of respondents

		(n = 12)	
Scale		Distribution	%
Sex	Male	6	50
	Female	6	50
Age	Mean (SD)	48.75 (10.6)	
	Range	30–68	
Discipline	Clinical ethicist	2	16.6
	Spiritual counsellor	3	25
	Medical specialist	3	25
	Paramedic	1	8.3
	Healthcare manager	2	16.6
	Nurse manager	1	8.3
Healthcare type	Hospital	6	50
	Mental healthcare	3	25
	Both	3	25
MCD methods used	Dilemma method alongside other methods	8	66.6
	Alternative methods	4	33.3

With the respondents’ consent, all interviews were recorded, transcribed and anonymized by the first author and an assistant. The VU University Medical Research Ethics Committee determined that the study does not fall under the requirements of the Medical Research with Human Subjects Act (*WMO*) as no actual interventions were performed.

### Data Analysis

The researchers aimed to identify the key elements of addressing worldview as part of MCD, based on facilitators’ personal experiences. Those experiences were defined as broadly and openly as possible using the grounded theory approach as developed by Charmaz (2006). The choice for grounded theory was made because we wanted to take the views and experiences of facilitators in moral case deliberation as a starting point in our research. The grounded theory approach implies not operationalizing the concept of worldview from a theoretical perspective beforehand. Data are collected by inviting respondents to present their own views and experiences and by subsequently analyzing this data.

Data analysis was carried out in three stages. The first stage involved open coding: the first two interviews were coded independently by two researchers and the results discussed by all three researchers. The topic list for subsequent interviews was then refined. The next two interviews were coded by the first researcher, after which the three researchers discussed the coding tree. The first researcher then conducted another eight interviews, two of which were co-coded by a research assistant.

During the second stage—focused coding—all codes were abstracted, overlapping themes and subthemes examined and their codes discussed by the first two researchers. This produced codes for 15 subthemes, formulated as gerunds or participles (verbs ending in ‘-ing’) in accordance with Tweed and Charmaz (Charmaz, 2006; Tweed & Charmaz, 2012). Gerund-based coding ensures a focus on actions rather than concepts, retaining a closer connection to the data (e.g., ‘devoting attention to different visions’ rather than just ‘different visions’). This approach suited our study since we sought to investigate how worldview is actually addressed in MCD practice.

The third phase—axial coding—examined the relationships among and patterns between the various themes, after which the over-arching themes and subthemes were refined and the final categories formulated. All authors agreed with the final set of categories, themes and subthemes.

## Results

This section describes the categories, themes and subthemes identified. The role of each theme in addressing worldview is discussed, as identified by both the respondents working with the dilemma method and those who favor other methods. A summary of the categories, themes and subthemes is given in Table 2.

**Table 2:** Summary of the key elements in addressing worldview

Category	Theme	Subtheme
Explicit role of worldview	Worldview of participants	Worldview of the patient
		Worldview of the professionals
	Approach of facilitators	Avoid stereotyping
		Devoting attention to different visions
Implicit role of worldview	Core values/inspiration behind values	Core values within the dilemma
		Professional inspiration
		Foundation of values
		Perspective of a good life
	Experiencing spiritual fulfillment	Fulfillment through connection
		The spiritual and existential dimension
	Lack of appropriate terminology	Difficulty of open discussion
		Embarrassment
	Approach of facilitators	Avoiding emphasis
		Thematization via norms and values

## Explicit Role of Worldview

The first category is concerned with the explicit role of worldview within MCD. This role is linked to clearly visible forms of religious beliefs or traditional belief systems. We first consider the worldview of the participants before discussing how the facilitators use this aspect to steer the discussion.

### Worldview of Participants

#### Worldview of the Patient

Worldview is relevant if it affects the specific case under discussion. This will certainly be the case where the dilemma involves patients with a clear religious background, such as practicing Jehovah’s Witnesses, Muslims or members of the Jewish community.

We have many patients with an Islamic background. We have also had Jehovah’s Witnesses on occasion, and have sometimes had to contend with the well-known dilemma of their unwillingness to accept blood transfusions. (8)

Because we were discussing the Jewish community, we considered the tragic situation of a woman who experienced particularly lengthy menstrual periods. It is not permitted to have sex during menstruation. Ovulation occurs after the onset of menstruation, so if you are not permitted to have sex during this period there is very little chance of conceiving a child. (9)

Moral standpoints can also be directly linked to the patient’s worldview, as illustrated by the following quote concerning attitudes to homosexuality:

I recently had a discussion about a patient of a mental health clinic somewhere in the eastern Netherlands. He is gay. His family had great difficulty accepting him, as did his fellow patients. Worldview certainly plays a part in this situation. (1)

#### Worldview of the Professionals

The worldview of professionals plays an explicit role within MCD if there is a conflict between professional responsibility and personal religion.

The dilemma might concern a nurse who is not willing to assist in certain interventions due to her worldview. (1)

So, in fact you’re being asked whether you would be kind enough to perform five abortions, bring five lives to a premature end, which we are supposed to find acceptable. The patient’s worldview has an effect on the entire nursing team. One member of that team is prepared to speak out. (11)

### Approach of Facilitators

#### Avoid Stereotyping

The first subtheme is the need to be aware of, and to avoid, stereotyping. If the situation is one in which worldview plays an explicit role, facilitators warn against the danger of stereotyping.

Of course, we consider the patient’s religious beliefs and how they affect what he considers important. You must be wary of falling back on stereotypes or preconceptions. (-) He would not wish treatment to be withdrawn. You really do have to be very careful not to jump to conclusions. (1)

I think that many preconceptions and prejudices are at play, whether about Christianity, Islam, Anthroposophy, or indeed any worldview that prompts you to place someone in a certain category. The danger is that any personal exchange about the values which underpin the worldview is overshadowed by the worldview itself. (5)

This can also happen because the facilitator omits to have the worldview explained by an MCD participant.

We have an analyst who is half Moroccan. And recently we have had dealings with some Moroccan couples. On one occasion there was an older gentleman who already had eleven children. He had a new, young wife and once again wanted to become a father. Due to his age, however—he was 80—his sperm was not up to the task. The analyst seemed to think that he was letting the Moroccan community down. I advised him not to think of himself as a representative of all Moroccans. We must also beware of allowing your personal vision of what it means to be Moroccan to prevail. (9)

#### Devoting Attention to Different Visions

The second subtheme is ‘devoting attention to different visions’. Respondents find it important for facilitators to address differences in worldview.

I think it is a very good thing when you look at those perspectives again and hear why someone is or is not willing or able to do something on the basis of their religion or other beliefs. I can appreciate that. It is laudable.(10)

It actually depends on my own idea of the case and what it is about. My vision of life, for example. You might believe that being alive is always a good thing provided there is no pain. A lot of people think that way. But there are also people who say that life is worth living regardless of whether there is pain. And even if someone is in pain, that’s not to say that they want to end their life. Pain is part of life. This represents a significant difference in worldviews and in people’s vision of life itself. (2)

## Implicit Role of Worldview

In the second category, we are concerned with the implicit role of worldview in the MCD process. In this category, worldview plays an implicit role in the background and is less clearly linked to world religions. It concerns the basis of core values investigated in MCD. Here, we first discuss core values and the inspiration behind them. The second theme is experiencing spiritual fulfillment. The third theme is the lack of appropriate terminology which would allow one’s worldview to be discussed openly, while the fourth theme is the question of how facilitators respond when implicit attention is devoted to worldview.

### Core Values and Inspiration Behind Those Values

#### Core Values Within the Dilemma

The first subtheme concerns the implicit presence of worldview in the core values relevant to the dilemma. In essence, core values are fundamental beliefs about what makes life valuable and worth living. They are, therefore, a part of the worldview. Devoting attention to core values creates awareness of what is important.

I always find it a sort of revelation when I realize why I stand for the things I do. I think it is wonderful (-) that you become self-aware like this – oh yes, I understand now. I do this because I believe that, and I find it extremely important. I live on the basis of my norms and values, so I do things in a certain way. (10)

It is actually the main consideration. (-) Yes, of course it’s about what you find important, what you consider worth pursuing. And it is about your own perspective of life. That might be a religious perspective or a secular one. It is all about worldview, nothing more or less. (1)

#### Professional Inspiration

The second subtheme is worldview as professional inspiration.

(-) and the other one says, ‘I have that at-home feeling’ I remember from nursing or whatever, why I actually do this work. I want the residents to have that same feeling. And he adds, I can’t remember finding that feeling so important.(5)

If I ask people about it, they say, ‘at last we have some opportunity to talk openly about our work and we can link it to the reasons we opted for this profession in the first place.’ In other words, we talk about inspiration, or the values and principles that are important to our work. (7)

#### Foundation of Values

The third subtheme is worldview as the foundation of values. The respondent indicates that worldview is the inside, the basis providing nutrition to values. Values are inspired by worldview.

We must then try to realize that the worldview is actually the inspiration to arrive at certain values. And it is those values which form the basis for further discussion. (4)

This can be difficult to talk about, because worldview is personal and less readily articulated, as is illustrated by the following quote:

I could say that values form the exterior of one’s worldview. If you ask about worldview, you are actually asking about the inner part behind the values. We do dare to say something about our values. They are the outer casing and they are in contact with each other. We are used to stating them. Those values are fed, and what feeds them is the inside part of the worldview. This is rather more personal because it is often less logical, less readily articulated. Some people can be embarrassed by their worldview. Perhaps it is not fully developed, or so full of dogmatic reasoning it is entirely inflexible. (12)

#### Perspective of a Good Life

The fourth subtheme is the worldview as the perspective of a good life.

I remember one MCD which I found particularly difficult. It was about an unborn baby who had been diagnosed as having a cleft lip and palate. (-) That is something that cannot be repaired completely but it is possible to bring about a significant improvement. Nevertheless, the parents were insisting that the pregnancy should be terminated. I asked the group to imagine that child playing with his friends, normal and intelligent in every way apart from that one little defect. What is a ‘good life’?

### Experiencing Spiritual Fulfillment

#### Fulfillment Through Connection

As the first subtheme in this category, respondents suggest that worldview is an implicit consideration in terms of shared spiritual fulfillment. This is recognized as the experience of a mutual connection between the participants in the MCD process.

MCD offers a way forward as well as an opportunity to speak openly and to reflect on an issue together, whereupon everyone has a much clearer idea of where we stand. I think it is also an opportunity for emotional processing, which may sound high-flown, but MCD should allow time and space for this. In this sense, it is cathartic for the participants. (3)

Worldview forms a prominent component of MCD because the participants experience it as a unique moment, for which the facilitator might even use a word such as ‘sacred’.

I think of these as truly sacred moments. (-) Something actually occurs… I think it is mainly the emerging connection, not only with each other but with the tragic situation. (2)

#### The Spiritual and Existential Dimension

The second subtheme is the spiritual and existential dimension of seeking the ‘right’ course of action.

… That is something I find almost spiritual – that MCD sets out to determine what I consider to be right and proper, the part I wish to play with regard to others, and whether I will actually be able to do so. (6)

But it is almost a sort of existential vision of the nature of reality. You’re saying that the world is not as it should be, whether by fault or design, so we can speak of a tragic situation. (-) You would need to be wearing blinkers to think that nothing is wrong. But the question is, how do we see precisely what is wrong? How do we describe the situation in words? Here, worldview plays a very significant implicit part, although in my experience it does not often manifest itself in an explicit way.(4)

### Lack of Appropriate Terminology

#### Difficulty of Open Discussion

The first subtheme in this category is the difficulty of discussing worldview due to the lack of a common terminology. Respondents indicate that in the current secularized society it is difficult to talk about worldview, because the religious language is no longer common.

I am convinced that worldview is a very important part of people’s lives, but my work has taught me that most people are unable to discuss their worldview fully because they cannot find the right words. For the same reason, it is difficult for me to broach the subject and I am reticent to do so. (12)

I would like to learn more about how worldview can be expressed in words, and this would probably be similar to the language we use to describe values. I hope that we will develop appropriate terminology together, and by ‘together’ I mean as a society. I see a certain linguistic paucity and helplessness, or at least clumsiness, when it comes to talking about abstract concepts such as worldview. Society as a whole has no common language, although certain groups such as religious communities have made moves in this direction. Nevertheless, the terminology remains fragmented and inconsistent. (12)

#### Embarrassment

The second subtheme concerns the embarrassment that people might feel when discussing matters of worldview.

Personally, I never inquire about someone’s worldview, perhaps because I sense a certain embarrassment, possibly due to the sheer difficulty of articulating very more abstract concepts, intuitions and ideas.(12)

Worldview in the general sense is sometimes brought up, but personal beliefs, religious or otherwise, are not. I get the impression that people find these matters too private to be discussed in an open setting such as an MCD group. As facilitator, one should probe and ask questions, but it would be wrong to embarrass participants or intrude in things they prefer to keep to themselves.(3)

During one recent MCD session, worldview was certainly raised by the person whose case we were discussing. ‘I am religious’, he told us. I did not ask him to explain further. Faith and religion are very broad terms. However, it felt almost like an admission of vulnerability. It is nevertheless important to understand why he opts to take a certain course of action.(9)

### Approach of Facilitators

#### Avoiding Emphasis

The first subtheme in this category is that the facilitator should avoid emphasizing worldview. There are various reasons for this. Doing so might, for example, hamper the discussion while some people may consider it inappropriate to talk about such matters in the hospital setting.

No, absolutely not because it really stands in the way of open discussion. If I announce that I am a protestant Christian, this creates all sorts of images in other people’s minds, none of which are likely to be particularly helpful. The other participants might jump to conclusions, or maybe I will suddenly think, ‘oh right, in that case I probably shouldn’t be in favor of euthanasia.’(6)

I would be very wary of doing so. (-) I’m mindful of being in the hospital setting, which is not really the place to seek philosophical depth. You are satisfied if people realize that you believe in your point of view and are happy to accept it. That is often enough. You might wish to pursue greater depth but I don’t really see that as my task. And given the time involved, it would not be appreciated. However, if you all want to enter a monastic retreat for a weekend and seek depth there, why not? That might be useful. (2)

#### Thematization via Norms and Values

Although facilitators generally avoid using the term ‘worldview’, they do investigate worldview aspects by asking about norms and values.

I never ask directly about worldview or religion, but I do enquire about what a person considers important. And I use that information. Someone whose worldview is based on anthroposophy, for example, might believe that nature should be allowed to take its course and medical interventions kept to a bare minimum. Muslims might object to the administration of morphine because ‘when you die, you must be able to look Allah in the eye.’ That is my approach – I always take norms and values into account. (5)

I do not ask about worldview to determine how a person sees a certain dilemma, but if we are discussing, say, euthanasia and someone says ‘no, I really couldn’t’, I find it useful to ask questions. What are the values on which he bases his objections?(10)

If people want to say something based on their worldview, that’s fine too. But I would not ask about worldview outright, at least not immediately. I would be more inclined to ask what particular values are important in this situation. (7)

A similar worldview can result in different values.

What most interests me about someone’s worldview is the values that are important within it. I can say that I am a protestant Christian, and perhaps you are too. But you may be a member of an entirely different church or denomination, or have an entirely different family background. As a result, your views about right and wrong may differ from mine. (6)

One respondent stated that worldview is examined during a session by means of general questions about the participants’ core values.

But what I often do is to go around the group and invite people to say a few words about the values they find important based on their upbringing. I might also ask what values they try to instill into their own children. These are often the person’s core values. (6)

## Discussion

Using the grounded theory approach, we investigated the role of worldview in MCD.

The grounded theory approach implies not operationalizing the concept of worldview from a theoretical perspective beforehand. For our purpose, we defined worldview as “fundamental beliefs about life, death and suffering that structure people’s ideas on how life events are related.” (Littooij et al., 2016a, p. 7). We have chosen this definition, because it is broad, inviting respondents to present their own views and experiences. The concept as defined is not opposed to current approaches in religious studies. Smart distinguishes 7 dimensions of worldviews: philosophical or doctrinal (beliefs), ethical, experiential, material, social, mythic and ritual (Smart, 1991). Our concept of worldview is broad enough to encompass these dimensions, but it refrains from explicitly addressing them during the interviews. Our results show that respondents address most of the dimensions distinguished by Smart, although the material and ritual dimensions are not present. A reason for this may be that MCD focuses on words and conversation, not on material objects or rituals.

The results reveal that worldview plays both an explicit and an implicit role.

Worldview becomes relevant in a number of specific examples, all of which are linked to clearly visible forms of religious belief. Respondents cite cases involving followers of the Islamic and Jewish faiths, as well as Jehovah’s Witnesses. The examples often involve some moral issue, such as objections to abortion, euthanasia or homosexuality. A conflict between religion and professional responsibility can arise in care givers who have such objections to some degree, whereupon the fulfillment of their professional duties results in a crisis of conscience. There may also be situations in which the professional is unable to accept or respect the patient’s views or beliefs.

Specific examples of religious worldviews can all too easily lead to assumptions based on stereotypes. Schweda et al. (2017) draw attention to the risk of stereotyping in end-of-life decisions, describing the variation and complexity of the relevant cultural and religious aspects. “There are no clear-cut positions anchored in nationality, culture or religion. Instead, attitudes are personally decided on as part of a negotiated context representing the political, social and existential situatedness of the individual.” (p. 1) The MCD facilitator should, therefore, devote attention to the various perspectives at play within the group and remain alert to any preconceptions that may exist in order to avoid the pitfalls of stereotyping.

Facilitators state that they consider it important to take the various visions into consideration. The respondents emphasize that worldview colors our moral beliefs. This bears out the findings of Turner’s (2003) study examining bioethics in a multicultural world. He notes that “…religious convictions and cultural norms play significant roles in the framing of moral issues” (p. 99). Turner also stresses the importance of taking the particular moral world of patients and their family members into account. Cultural and religious traditions determine how people view birth, illness, suffering and death. A more anthropological approach to ethical issues can help to raise awareness of the role of culture and religion in MCD (Turner, 2003).

Worldview also plays an implicit role, being the basis of core values investigated in MCD. Those core values represent fundamental beliefs with regard to the value of life: what makes life worth living? Careful discussion of the core values can therefore help MCD participants to identify the crux of the issues at hand (Widdershoven et al., 2016, p. 73, 79, Hartman et al., 2016, p. 78).

One specific area in which worldview (in the form of core values) can further the MCD discussion is the professional inspiration of caregivers. Rushton (2017) points out that keeping sight of one’s original motivation for practicing a certain profession helps to promote resilience (Rushton, 2017) and the ability to function well. According to Geller et al. (2008), motivation includes the desire to be of significance to the patient.

Worldview is also seen as the inner part and inspiration behind values. It thus is tangent to the base of values and displays the foundation on which values are grounded. Worldview shows the fundamental nature of values. In order to clarify the fundamental nature of values, more explicit attention for worldview might be useful during MCD and contribute to the deliberation. We would advise facilitators to be alert to statements or terms which may reveal something about the speaker’s worldview (Alma, 2008, p. 62). However, facilitators point out the difficulty in discussing this inspiration, which involves matters which are sensitive and do not lend themselves to verbal expression.

During the MCD process, participants attempt to identify what constitutes ‘a good life’. By encouraging explicit discussion of this topic, facilitators can thematize worldview. Doing so will also make participants more aware of their reasons for making choices.

MCD is also beneficial in that it can bring about fulfillment through connection and touch upon the spiritual and existential dimension of ethical issues. The element of connection implicitly refers to *religion*, in the sense of the Latin *religare,* one meaning of which is ‘to bind together’. This implies both the connection with important topics and the connection with each other. The spiritual and existential dimension touches upon hope, inspiration and healing (Alma, 2018). The relationship between worldview and healing shows marked similarities with that between worldview and coping (Pargament & Ano, 2006; Körver, 2013; Balboni et al., 2007; Puchalski et al., 2009). The literature on the relationship between worldview and coping notes that, next to support by talking, support based on rituals can be effective. Rituals can enhance social cohesion and the ‘sense of community’ (Ladd & Spilka, 2013, p. 445). Perhaps the steps of MCD can themselves be regarded as creating a ritual which may enable participants to deal with difficult moral issues in life.

Addressing worldview is not a simple matter, for various reasons. There is no common language to describe the various aspects involved, and the use of a ‘high-blown’ term such as worldview may itself cause some embarrassment. These limitations account for the changing position of worldview—and in more general terms, religion—in today’s society. Under the influence of modern rationalism, existential themes have been banished to the private sphere. In the public domain, people are more concerned with understanding the causes and effects of more concrete phenomena (Vanheeswijck, 2008) rather than ‘the final questions’ (Alma, 2018, p.53).

Bauman and Donskis (2013) suggest that there is growing reticence to discuss worldview, and a gradual loss of appropriate terminology, due to secularization and individualization. The search for moral and spiritual significance is increasingly a solo undertaking (Alma, 2018, p. 54). The disappearance of institutionalized, organized worldviews with moral, existential and spiritual authority in western society means that there is no longer a common language which would enable people to talk to each other about their vision of a good life, or to reflect upon the social constructs which could inform their actions and decisions. (Alma, 2018, p. 54).

Worldview is an implicit component of any discussion about norms and values. Values can form a starting point for a reflection on what is valuable and worthwhile in life. Here, we must ask whether a more explicit use of the term ‘worldview’ would increase the cohesion of the various values within someone’s vision of ‘a good life’, thus furthering their thematization. We propose to further explore this potential addition to the MCD methodology, as has been done regarding the explicit thematization of emotions (Molewijk et al. 2011a, b).

### Strengths and Limitations

As far as we know, this is the first study examining the visions and experiences of facilitators on addressing worldview in MCD. Our study, however, has some limitations. The interviews were conducted by a researcher with a background in pastoral care. This may have influenced the interviews. A second limitation is that the study was conducted in the Netherlands, in a largely secularized society. This may limit generalization to other countries. A third limitation is that the interviews were held with facilitators. Interviews with MCD participants might give information about their experiences and complement the results.

## Conclusion

According to the facilitators taking part in this study, worldview plays both an explicit and an implicit role in the MCD process. The explicit role concerns the religious beliefs of patients and professionals. This calls for alertness in order to avoid stereotyping. The implicit role involves the core values, intentions and inspiration of the participants. Aspects of worldview are also at play in the creation of connection between participants, and their experience of the spiritual and existential dimension of ethical dilemmas. In order to clarify the fundamental nature of values, more explicit attention for worldview might contribute to the deliberation. Including aspects of worldview might enhance the methodology of MCD, allowing greater opportunity for reflection on aspects for which appropriate terminology is lacking in our modern society. However, this should be done with caution as the term ‘worldview’ might be interpreted by participants in terms of religious and personal beliefs, rather than as an invitation to reflect on one’s view of the good life as a whole.

## Data Availability

The dataset that supports the conclusion of the article is available in Dutch.
